# A Single-Center Early Experience of Endoscopic Submucosal Dissection for Gastric Lesions in Thailand

**DOI:** 10.1155/2020/6873071

**Published:** 2020-01-30

**Authors:** Prasit Mahawongkajit

**Affiliations:** Department of Surgery, Faculty of Medicine, Thammasat University, Pathum Thani, Thailand

## Abstract

Endoscopic submucosal dissection (ESD) was also considered a “high risk” at the starting point with skillful endoscopic techniques and terrible complications. Still, it remains challenging yet has become widespread among institutes in many parts of the world. This study is aimed at further investigating the feasibility, efficacy, and safety of ESD of gastric lesions and at evaluating clinical outcomes in early experience. The patient characteristics, postoperative outcomes, and results of histopathological examinations were reviewed retrospectively between January 2017 and May 2019. Thirteen patients' gastric ESD were included with all of en bloc resections without recurrence. The long duration was related to the large lesion, the upper part of the stomach, and previous treatment. In this study, the ESD procedure of the stomach is a feasible and safe minimally invasive treatment option with organ preservation. It requires training and experience with a learning approach where skill may be improved to prevent unwanted complications.

## 1. Introduction

Endoscopic resection is one of the accepted methods for treating early gastric cancer [1–3]. Endoscopic mucosal resection (EMR) has a disadvantage: to ensure complete resection and to gather accurate pathological information, usually for the lesion greater than 20 mm, resection is performed piecemeal and may be associated with local recurrence [4]. Endoscopic submucosal dissection (ESD) was first reported in the late 1990s in Japan [5]. ESD has the most crucial advantage for en bloc complete resection possible, especially for large lesions [4, 6]. The method of ESD was performed for lesions with a negligible risk of lymph node metastasis [7, 8] at the mucosa and submucosa. Also, ESD could be applied as a therapeutic and diagnostic tool for gastric lesions in cases where the forceps biopsy pathology is discrepant from the endoscopic findings [9].

Despite these interesting advantages, ESD was also considered a high risk at the starting point with skillful endoscopic techniques and terrible complications such as perforation and bleeding [10, 11]. However, ESD remains challenging, and it has become widespread reaching institutes in many parts of the world by hands-on workshops with *ex vivo* and *in vivo* animal training models under the supervision of ESD masters [12–20]. The current study is aimed at further investigating the feasibility, efficacy, and safety of ESD of gastric lesions and at evaluating clinical outcomes with respect to the learning curve of early experience.

## 2. Patients/Material and Methods

This study was conducted as a retrospective review with the patients referred to the surgical department with treated gastric lesions by ESD between January 2017 and May 2019 and identified in the hospital electronic documentation system. This study passed the ethical research process from the Human Ethics Committee of Thammasat University (Faculty of Medicine) with a reference number: MTU-EC-SU-0-076/62.

The patients who had a gastric lesion underwent careful endoscopic evaluation (GIF-H290Z; Olympus Corporation). The morphological endoscopic classification was made according to Paris classification [21] using white light, narrow-band imaging (NBI), chromoendoscopic technique with indigo carmine dye, and magnifying endoscopy. If the gastric lesion prompted doubts about the possible deep invasion, complementary endoscopic ultrasonography (EUS) staging was performed. All gastric lesions underwent endoscopic biopsies to confirm their pathology. The patients were informed of the treatment options and consented to undergo ESD.

**Figure 1:**
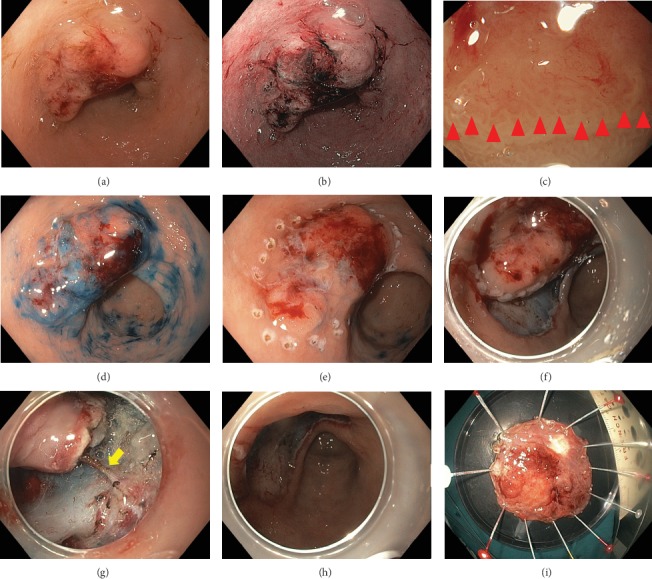
Endoscopic submucosal dissection (ESD) procedure. (a) Endoscopic view of 28 mm 0-IIa lesion at lesser curvature, lower part of the stomach. (b) Narrow band imaging. (c) Demarcation line (red arrow) with abnormal microvascular and microsurface by magnified endoscopy. (d) Chromoendoscopy with indigo carmine dye. (e) Completed circumferential endoscopic marking for resection margin of lesion. (f) Endoscopic mucosal incision and submucosal dissection. (g) A submucosal vessel was identified (yellow arrow) and hemostasis by Coagrasper hemostatic forceps. (h) Resection area after dissection and hemostasis. (i) Resected specimen of gastric lesion.

ESD was performed as follows: The patient was placed under general anesthesia in a left lateral decubitus position. Flexible endoscopy (GIF-HQ190; Olympus Corporation) was performed using a flexible overtube (MD-48518; Sumius; Sumitomo Bakelite Co., Ltd., Tokyo, Japan). The lesion was carefully inspected to determine the precise margins. Subsequently, multiple mucosal markings were generated around the lesion using a dual knife (KD-650L; Olympus Corporation, Tokyo, Japan). The injection solution was prepared with 200 ml Glyceol (glycerin 10%, fructose 5%, and NaCl 0.9%) and 1 ml of indigo carmine dye. The lifting solution was endoscopically injected into the submucosal layer adjacent to the lesion. The lesion was subsequently completely resected and removed via endoscopic circumferential mucosal incision and careful submucosal dissection using a dual knife, a flexible endoscope with soft straight distal attachment at tip of the scope (D-201-11804; Olympus Corporation, Tokyo, Japan), and a VIO300D device (Erbe Elektromedizin GmbH, Tübingen, Germany). Control of bleeding was obtained with coagulation via a dual knife and Coagrasper hemostatic forceps (FD-410LR; Olympus Corporation, Tokyo, Japan) (Figure 1).

All procedures were done by a single operator who had experience in performing over 1,000 endoscopic procedures, had attended and discussed live cases with expert Japanese ESD masters over 400 cases, had practiced on an ex vivo model, and completed gastric ESD training course on live animal models without complication. The data recorded the patient characteristics, ESD procedures, postoperative courses, the results of the histopathological examination, and short-term outcomes.

## 3. Results

Thirteen patients with gastric lesions who were informed of the therapeutic procedures and consented to undergo ESD were included in this study (Table 1). The mean age was 62.6 (range 52 to 79). There were two males and 11 females. Only one had received prior therapy with EMR. The majority of the lesions (53.85%) were located in the lower third of the stomach. The macroscopic type of lesion, based on the Paris classification, was 0-I found in more than half the lesions without ulceration. The average size of the lesion was 27.5 mm.

The mean operative duration was 145.5 minutes over all of en bloc resection. For adverse events, post-ESD bleeding developed within 72 hours in one patient. The endoscopic examination demonstrated a bleeding vessel at the base of the resected part, which was treated with epinephrine injection and electrocautery. The mean postoperative hospitalization was 5.2 days. The final pathological diagnosis of gastric ESD was adenoma (*n* = 2), low-grade dysplasia (*n* = 3), high-grade dysplasia (*n* = 4), and adenocarcinoma (*n* = 4). In the gastric adenocarcinoma group, the depth of invasion was intramucosal (*n* = 1), in the submucosal layer with less than 500 *μ*m (*n* = 2) and more than 500 *μ*m (*n* = 1). The mean follow-up for patients was 502 days (range, 1-767) without recurrence in all patients (Tables 2 and 3).

## 4. Discussion

ESD is a standard treatment in Japan and became a recommendation in other countries for early gastric cancer worldwide. EMR of lesions larger than 20 mm has a piecemeal resection style due to technical limitations, giving rise to unsatisfactory pathological results and increased recurrence. ESD has the advantage of nullifying these by en bloc resection, especially for large lesions to make satisfactory pathological staging along with potential therapeutic success. Nowadays, gastric ESD has been further used for premalignant epithelial lesions and the role of lesions where pathologic findings are at odds with the endoscopic interpretations in terms of clarifying the final pathologic diagnosis.

Although ESD is a novel, challenging technique, it carries the risks of serious complications, long procedural duration requiring substantial training, and expertise development. ESD is still gradually expanding worldwide over recent years to improve the quality of treatment modality for gastric lesions [6, 12–20]. In this retrospective report, the patients underwent an ESD procedure for gastric lesions, and their characteristics, postoperative courses, the results of the histopathological examination, and outcomes were analyzed. The mean diameter of the lesion was 27.5 mm without ulceration. All resected specimens were completed en bloc resection without recurrence. For early gastric cancer in Thailand, the incidence is low, and this study reported four patients underwent ESD following the Japanese gastric cancer treatment guidelines [2]. The final pathological report showed adequate endoscopic treatment in one intramucosal case and two cases of lesions that invade the submucosa less than 500 *μ*m. One patient was diagnosed with adenocarcinoma that invades into the submucosa more than 500 *μ*m proceeding to surgery. The final pathology of laparoscopic-assisted distal gastrectomy demonstrated no residual tumor and negative for all lymph node dissections.

The average duration of the procedure was 145.5 minutes. The factors that related to longer procedural time were large lesions, the upper part of the stomach, and scarring from previous EMR. The duration of five gastric ESD having lesions located at the upper part showed longer than the average procedural time (140, 155, 168, 170, and 317 minutes). The technical demands of ESD are more difficult in the upper part due to bending and the retroflexion position of the endoscopy. The previous EMR had created fibrotic scarring, and it presented more difficulty in the step of submucosal dissection. However, the experience of ESD procedures in this study may be taken as rather a small number.

Regarding complications, one patient had an adverse event. The patient received ESD for an intramucosal lesion with high-grade dysplasia. She had been treated with aspirin and clopidogrel for underlying heart disease and had discontinued before the procedure. The 0-IIa gastric lesion was removed from the greater curvature of the middle part within 70 minutes. On the third day of postoperation, the patient developed bleeding, and reendoscopy was done. The point of bleeding was detected from the base of the resected area and stopped. Procedure-related bleeding may be associated with factors, including age and anticoagulant drugs [22]. The patient was discharged on the 7th day without other complications and did not require blood transfusions.

Currently, ESD is the advanced, minimally invasive procedure that allows for en bloc curative resection of superficial lesions of the stomach and provides pathological data for proper management. ESD is technically demanding and may be associated with severe complications. It requires training and experience with a learning attitude that may prove skill and prevent unwanted complications. In this study, the ESD procedure of the stomach is a feasible, safe, minimally invasive treatment option with organ preservation. This study is an early experience with a small number of patients. Further studies are required to reassure that ESD can be encouraged as the standard treatment for superficial gastric lesions in Thailand.

## Figures and Tables

**Table 1:** The patient and lesion characteristics that were treated by endoscopic submucosal dissection.

Procedure, n	ESD^#^ = 13
Age, mean ± SD (years)	62.6 ± 7.1
Sex, male/female	2/11
Macroscopic, *n* (%)	
0-I	7 (53.85)
0-IIa	4 (30.77)
0-IIb	1 (7.69)
0-IIc	0 (0)
0-III	0 (0)
Mix	1 (7.69)
Location, *n* (%)	
Upper third	5 (38.46)
Middle third	1 (7.69)
Lower third	7 (53.85)
Position, *n* (%)	
Anterior wall	4 (30.77)
Greater curvature	4 (30.77)
Posterior wall	1 (7.69)
Lesser curvature	4 (30.77)
Lesion size, mean ± SD (mm)	27.5 ± 8.7
Lesion with ulceration, *n* (%)	0 (0)
Prior treatment, *n* (%)	
EMR^##^ only	1 (7.69)
Combination (EMR, APC^###^)	0 (0)

^#^ESD: endoscopic submucosal dissection; ^##^EMR: endoscopic mucosal resection; ^###^APC: argon plasma coagulation.

**Table 2:** Outcomes of endoscopic submucosal dissection for gastric lesions.

Procedure	ESD^#^ = 13
Operative duration, mean ± SD (minutes)	145.5 ± 61.8
En bloc resection, *n* (%)	13 (100)
Incomplete resection, *n* (%)	0 (0)
Size of lesion (mm)	27.5 ± 8.7
Size of resection specimen (mm)	31.8 ± 10.4
Specimen area/lesion area ratio	1.16 ± 0.06
Postoperative hospitalization, mean ± SD (days)	5.2 ± 0.9
Adverse events, *n* (%)	1 (7.69)
Early (within 24 hours), *n* (%)	
Bleeding	0
Perforation	0
Late, *n* (%)	
Bleeding	1 (7.69)
Perforation	0
Stricture	0
Pathological diagnosis, *n* (%)	
Adenoma	2 (15.38)
Adenocarcinoma	4 (30.77)
Intramucosal	1 (7.69)
SM^$^ < 500 *μ*m	2 (15.38)
SM^$^ ≥ 500 *μ*m	1 (7.69)
Lymphovascular invasion	0 (0)
Low-grade dysplasia	3 (23.08)
High-grade dysplasia	4 (30.77)
R0 resection	13 (100)
Recurrence, *n* (%)	0 (0)
Survival, *n* (%)	13 (100)
Mean follow-up (days)	502 ± 236

^#^ESD: endoscopic submucosal dissection; ^$^SM: submucosa.

**Table 3:** Details of 13 patients treated by endoscopic submucosal dissection.

Patient number	Age (years)	Sex	ASA	Macroscopic	Location	Tumor size (mm)	Ulceration	Procedure time (minutes)	Pathological diagnosis	R0	Depth	Adverse events	Hospital stay (day)	Recurrence	Survival	Remarkable
1	58	Female	1	0-IIa	Lower, greater curvature	24	No	118	Low-grade dysplasia	Yes	Intramucosal	No	4	No	Alive	
2	62	Female	1	0-I	Lower, anterior wall	27	No	135	High-grade dysplasia	Yes	Intramucosal	No	5	No	Alive	
3	69	Male	2	0-I	Lower, anterior wall	25	No	120	Adenocarcinoma	Yes	Intramucosal	No	6	No	Alive	
4	68	Female	1	0-IIb	Lower, posterior wall	25	No	130	High-grade dysplasia	Yes	Intramucosal	No	5	No	Alive	
5	57	Female	1	0-I	Upper, anterior wall	22	No	168	Low-grade dysplasia	Yes	Intramucosal	No	5	No	Alive	
6	79	Female	1	0-IIa	Middle, greater curvature	28	No	70	High-grade dysplasia	Yes	Intramucosal	Bleeding on the 3^rd^ day	7	No	Alive	
7	63	Female	1	0-I with scar (previous EMR)	Lower, lesser curvature	30	No	185	Adenocarcinoma	Yes	Submucosa (<500 *μ*m)	No	6	No	Alive	
8	53	Female	1	0-I	Upper, posterior wall	20	No	170	Adenoma	Yes	Intramucosal	No	4	No	Alive	
9	65	Female	2	0-IIa	Lower, lesser curvature	28	No	105	Adenocarcinoma	Yes	Submucosa (≥500 *μ*m)	No	6	No	Alive	Surgery
10	65	Female	1	0-IIa	Upper, greater curvature	26	No	155	High-grade dysplasia	Yes	Intramucosal	No	4	No	Alive	
11	63	Female	1	0-I	Upper, greater curvature	22	No	140	Adenoma	Yes	Intramucosal	No	4	No	Alive	
12	60	Male	1	0-I	Upper, lesser curvature	55	No	317	Adenoma	Yes	Intramucosal	No	6	No	Alive	
13	52	Female	1	0-IIa+0-IIc	Lower, lesser curvature	25	No	78	Adenocarcinoma	Yes	Submucosa (<500 *μ*m)	No	5	No	Alive	

## Data Availability

The patient data used to support the findings of this study are restricted by the Human Ethics Committee of Thammasat University (Faculty of Medicine) in order to protect patient privacy. Data are available from Prasit Mahawongkajit (prasit_md@yahoo.com) for researchers who meet the criteria for access to confidential data.
